# The role of Bob1 in rheumatoid arthritis: potential implications for autoimmunity

**DOI:** 10.1186/1479-5876-10-S3-O5

**Published:** 2012-11-28

**Authors:** Nataliya Yeremenko, Tineke Cantaert, Melissa N van Tok, Ioana Gofita, Juan D Canete, Paul-Peter Tak, Hergen Spits, Dominique L Baeten

**Affiliations:** 1 Division of Clinical Immunology and Rheumatology and Dept. of Experimental Immunology, Academic Medical Center / University of Amsterdam, Amsterdam, The Netherlands; 2 Dept. of Rheumatology, Hospital Clinic, Barcelona, Spain; 3 Tytgat Institute for Liver and Intestinal Research; Academic Medical Center / University of Amsterdam, Amsterdam, The Netherlands

## Background/purpose

Rheumatoid arthritis (RA) is a prototypic autoimmune disease characterized by a prominent humoral autoimmunity. Of particular relevance is the local production of autoantibodies such as rheumatoid factor and anti-citrullinated protein antibodies in the inflamed synovial tissue. The mechanisms underlying break of B cell tolerance and local autoantibody production remains poorly understood. This study was conducted in order to identify cellular and molecular pathways implicated in RA-specific humoral autoimmunity.

## Methods

Synovial tissue samples were obtained by arthroscopy from untreated individuals with RA (n=33) and inflammation matched SpA controls (n=58). Gene expression profiling was performed on tissue samples of patients with established arthritis using 44K Whole Genome Human microarrays (Agilent). Top differentially expressed genes were validated on three independent cohorts by Taqman based RT-qPCR and immunohistochemistry. Collagen-induced arthritis (CIA) and Experimental autoimmune encephalomyelitis (EAE) experiments were conducted using Bob1 knockout mice and their littermate controls.

## Results

Microarray screening for genes differentially expressed in the inflamed synovium, the key target of the disease process in RA, revealed a prominent and disease-specific B cell/plasma cell signature with the B cell-specific transcriptional co-activator Bob1 and its transcriptional target BCMA among the most upregulated genes. Validation by RT-qPCR on two independent cohorts representing early and established arthritis confirmed microarray data and demonstrated elevated expression of Bob1 and BCMA not only in established RA, but also at the early phase of the disease. Quantitative evaluation of immunohistochemical stainings of synovial tissue with monoclonal antibody for Bob1 revealed significant increase in Bob1 positive cells in RA synovium (p<0.01). Next we determined whether lack of functional Bob1 modifies disease onset or severity in CIA. Interestingly, the results showed that Bob1^−/−^ mice were fully resistant to CIA induction compared to their wild-type littermates. This remarkable protection from CIA is explained by failure to produce pathogenic anti-collagen autoantibodies in the absence of Bob1. In contrast, Bob1^−/−^mice were susceptible to MOG protein induced EAE and incidence and severity of clinical disease were not altered in these mice comparing to wild-type littermates, suggesting that absence of Bob1 does not impact on antigen-presentation/costimulatory capacity of B cells.

## Conclusion

The specific increase in Bob1 expressing cells in RA synovitis and the resistance of Bob1-defecient mice to development of CIA indicate that Bob1/BCMA axis may contribute to humoral autoimmunity in RA. The relationship between an aberrant Bob1 expression and the break of peripheral tolerance in RA is currently under investigation.

